# Ageism, an invisible social determinant of health for older Syrian refugees in Lebanon: a service providers’ perspective

**DOI:** 10.1186/s13031-022-00491-9

**Published:** 2022-11-24

**Authors:** Maya Abi Chahine, Hanna Kienzler

**Affiliations:** 1 Faculty of Health Sciences, American University of Beirut, Bliss Street, Beirut, Lebanon; 2 Department of Global Health and Social Medicine, ESRC Centre for Society and Mental Health, King’s College London, Bush House (NE) 3.15, 40 Aldwych, London, WC2B 4BG UK

**Keywords:** Older refugees, Ageing, Ageism, Social determinants of health, Syrian refugees, Syrian war, Social isolation, Non-communicable diseases, Mental health

## Abstract

**Background:**

Older refugees face particular challenges because their health and social needs are largely overlooked in humanitarian programmes, policies and research. The few studies available have shown that older refugees suffer from a high prevalence of non-communicable diseases, including mental health problems, increased social isolation and poverty, and difficulty accessing health and social services. This article aims to provide further in-depth understanding of how service providers perceive health and social challenges of older Syrian refugees living in Lebanon by focusing on (1) their health and social challenges; (2) the available and lacking services; (3) participation; and (4) policy recommendations to improve services.

**Methods:**

This study is based on a qualitative research approach. Fifteen semi-structured interviews were conducted with health and social workers providing services to older Syrian refugees living in Lebanon. All interviews were digitally recorded, transcribed, coded and analysed using thematic analysis.

**Results:**

Study results clearly show that older refugees face increased marginalisation and neglect, mainly because of ageism. Ageism experienced at aid agency, family and individual levels, impacts negatively on older refugees. They have a sense of social isolation, neglect and feel they are a burden, consequently their social participation decreases, impacting negatively on their physical and mental health as well as their access to social and health care. Linked to experiences of ageism, study participants noted: (1) high prevalence rates of non-communicable diseases and mental health problems; (2) difficulties accessing care, with inadequate services to support the needs of older refugees; and (3) policy recommendations calling for an holistic approach to aid which takes into consideration the specific needs of older refugees as well as their capabilities.

**Concluding remarks:**

Ageism is a key determinant of health which negatively impacts the physical, mental and social health, and wellbeing of older Syrian refugees. It pushes them to the margins of society where they are left behind by the humanitarian response, policy makers and researchers, as well as their communities and families. To mitigate this situation, this article calls for directly addressing ageism on social, service and policy levels.

## Introduction

This article provides insight into the health and social challenges faced by older Syrian refugees from the perspective of service providers in Lebanon. Their situation is interlinked with the global refugee crisis, which has been described as the biggest since the Second World War. The latest estimates by UNCHR suggest there were 80 million forcibly-displaced people worldwide, including 26.3 million refugees, in 2021 [1]. It is further highlighted that 67% of the refugees originate from only 5 countries, with Syria in the lead. Developing regions host 86% of the world’s refugees under UNHCR’s mandate, with Turkey hosting 3.6 million Syrian refugees, Lebanon hosting 1.5 million Syrian refugees, and Jordan 666,692 [1–3]. Among these refugees, older people (aged 60 years and older) constitute around 4% under UNHCR mandate [4]. This number is believed to reflect under-reporting as older refugees tend to be “significantly under-registered (as refugees) compared with other groups” [5], p. 16]. Reasons behind under-registration of older refugees compared to refugees from other age groups mainly relate to difficulties reaching registration sites due to both physical and financial reasons and also lack of information about registration process, lack of trust in the benefits they would receive and fear of having their names recorded on official documents [5]. Moreover, older people are usually the last to flee their countries in times of crisis due to physical, financial and emotional reasons [4, 6].

Lebanon has the largest refugee population per capita in the world [3]. The numbers have significantly increased following the start of the Syrian conflict in 2011. As a consequence, public services, which were already fragile, were soon considered exhausted. Older refugees are particularly affected by these challenges in terms of their livelihoods, economic situation and health. Despite this insight, the needs of older refugees continue to be overlooked, not only in humanitarian programmes, policies and research in Lebanon, but globally [7]. For instance, HelpAge International has found that only 5.3% of analysed aid projects mentioned older people as a vulnerable group, and less than 1% of humanitarian funding addressed their needs [5, 8]. Moreover, policymakers and service providers in host countries have been blamed for putting older refugees at the bottom of their refugee aid agendas [6, 9, 10]. At the same time, it is increasingly recognised that investigating and responding to the needs and priorities of older refugees is important amidst the world-wide trend of rapid ageing and related new health risks and needs which are believed to be even more challenging in emergency settings [11–13]. There is thus a marked discrepancy between the heightened need to provide support to older refugees and the lack of dedicated resources and data to do so effectively.

This discrepancy has been linked to ageism among aid providers, governments and local communities. Ageism is defined as “the systematic stereotyping and discrimination against people, simply because of their age” [14], p. 55]. Despite the fact that research has shown older refugees are systematically excluded from aid services leading to social isolation, neglect, feelings of being a burden and a consequent decrease in their social participation [2, 15–19], only scarce information is available on ageism in humanitarian settings [11].

### Health and social challenges faced by older refugees

What we know about older refugees in emergency settings and host country contexts worldwide is based on a few case studies published in academic journals and humanitarian aid agency reports [20]. The findings from the available literature outlined below highlight the main health and social challenges as well as particular access barriers to health and social services and community participation.

Reports have shown that older refugees struggle to access basic needs including water, food and shelter due to lack of mobility, social isolation and poverty which puts them at increased risk of illnesses, bad hygiene and malnutrition [9, 11, 21–23]. The most common physical health problems among older refugees are non-communicable diseases (NCDs) such as diabetes, stroke and cancer [5]. For example, a study carried out by HelpAge International and Handicap International found that 54% of older Syrian refugees in Jordan and Lebanon suffered from at least one NCD, while others suffered from more than one [[5], see also [24]]. Mental health and psychosocial problems are also highly prevalent among older refugees, with 50% self-reporting depression [12, 22]. This high prevalence is thought to be due to older refugees being more attached to their homeland, suffering from losing social support and status, and having no future prospects. Additional stressors negatively affecting the mental and psychosocial health of older refugees include key social determinants of health such as poverty, inappropriate housing, insufficient food and family disintegration [9, 25–27].

NCDs have been shown to be particularly challenging to treat in refugee settings due to their chronic nature, difficulty in diagnosing, high treatment costs, and the requirement for continuous care and medication which is often unavailable for refugees [28]. Moreover, available health services are difficult to access for older refugees because of barriers which are as much physical (e.g., non-age-friendly healthcare facilities), as attitudinal, with older people’s health and wellbeing being considered less pressing compared to younger people. In Bosnia, for example, chemotherapy was made available exclusively to children despite a much higher prevalence of cancer among older refugees [11, 23].

Such barriers have been linked to ageism and this further exacerbates the difficult situation of older refugees as it leads to social isolation and lack of participation [29]. For instance, in Tanzania, younger adults requested older refugees to leave the camp accusing the latter of depleting vital resources [23], while in Ukraine, 55% of older refugees seeking assistance were turned down by humanitarian aid providers based on their age [30]. In both contexts, older refugees were more at risk of social isolation and loneliness, and rarely participated in social and civic activities. Conversely, social participation has been shown to be particularly important for older people’s health and mental wellbeing [29]. While older refugees are often portrayed as passively benefiting from assistance, they are in fact an invaluable resource to their families and communities passing on traditional conflict resolution skills to the young, handing down cultural tradition and values, and, in several cases, taking the lead in returning to their home countries following wars and conflict [9, 11, 31–33].

### Health and social challenges of older Syrian refugees in Lebanon

Our literature search about health and social challenges faced by older Syrian refugees in Lebanon revealed only two academic articles and two reports, with data dating back to 2013 and 2015 [5, 34–36]. Insights from these four studies are based on relatively small sample sizes ranging from 210 to 91.

The studies revealed that NCDs are the most prevalent health problems among older Syrian refugees with 66% of them suffering from at least one NCD [34, 35], 33% suffering from severe impairment and 60% having problems in activities of daily living [5]. Mental health was another grave concern with 65% of older Syrian refugees suffering from psychological distress [5], mainly due to physical health problems, poor living conditions, loss of social connections and a feeling of being a burden [34, 36]. Healthcare was mainly organised through UNHCR which covered registered refugees for Primary Health Care (PHC) against a reduced fee for consultations, and with free diagnostic tests for those above 60 years of age. Secondary and tertiary care were limited to life-threatening diseases with only 75% of the cost covered [37–39]. Overall, 79% of older Syrian refugees were reported to have problems accessing healthcare, and only 1.5% could afford it [30]. Mental health service provision appeared to be largely missing due to a lack of political will to make them available to the older refugee population.

The literature highlighted that daily stressors further exacerbated health problems. Shelter was pinpointed as the major challenge for older Syrian refugees. In 2013, 48% of older refugees lived in tents, unfinished structures or other dwelling sites which were precarious and dangerous [34]. Precarious shelter conditions affected older refugees whose health and safety requires easy access to water and latrines to prevent their falling due to obstacles, and proper heating to prevent illnesses. Moreover, employment and poverty were listed as major daily stressor [40]. In 2012, only 2% of older Syrian refugees were able to provide for their needs, 74% relied on aid and 22% on family. They suffered from lack of food, medication and financial support [34–36].

Given the clear gap in literature related to the experiences of older Syrian refugees in Lebanon our research aimed at focusing on the following questions: What are the main health and social challenges faced by older Syrian refugees in Lebanon’s camps and host communities from the perspective of health and social workers? What are the available and lacking services from the service providers’ perspectives? How do health and social workers perceive the vulnerability and participation of older Syrian refugees? What are the policy recommendations of service providers to promote inclusion of older persons in humanitarian aid in order to better serve their specific needs?

## Methods

This study followed a qualitative approach to investigate the health and social problems faced by older Syrian refugees in Lebanon from the perspective of health and social workers. Particular attention was paid to how service providers viewed older Syrian refugees’ physical health and mental health, with particular focus on social determinants of health, access to support and care, and suggestions for improvement of policy and practice in this field. The qualitative research design allowed interpretation of issues under investigation based on the meanings given to them by study participants, plus in-depth examination of sensitive topics, looking into their behaviour, beliefs and attitudes [41–44]. The study was carried out in Lebanon between May 29 and June 20, 2018. Ethical approval was obtained from King’s College London’s Research Ethics Board (MRS-17/18-6337) prior to recruitment and data collection.

The first author recruited a purposive sample of health and social workers from five NGOs working directly with older refugees in refugee camps and host communities in Lebanon. NGOs and interviewees were chosen to provide diverse accounts of services provided in different areas of the country. Three of the NGOS were Lebanese and two were international. They operated in various governorates: Beirut, South, North and Bekaa Valley. Only one NGO offered health services and another only social services, while the others delivered a combination of health and social services. From these NGOs, a total of fifteen interviewees were recruited including eleven women and four men from their mid-twenties to 60 s. We chose interviewees from diverse specialties to cover a wide array of services: two counsellors, two general practitioners (GPs), two mental health nurses, two nurses, two psychologists, and five social workers. All had been working with older Syrian refugees since the beginning of the Syrian civil war in 2011 except for four who had worked in the field for between eighteen months and three years.

Sampling followed a purposeful and snowball sampling approach. The first author contacted potential participants by email, inviting them to the study and providing the information sheet. After three days, she followed-up by telephone to further explain the aims and answer any questions potential participants might have. Once the participants agreed to take part in the research, they were provided with the consent form which they were asked to fill out and sign prior to the interview. These participants were then asked to connect the first author with colleagues working in their NGO as well as in other aid agencies, who were then recruited in the same manner as outlined above.

Semi-structured interviews were conducted with all study participants. The interview guide inquired into (a) the main health and social challenges of older refugees; (b) services, resources and barriers to care access; and (c) policy recommendations. All interviews were conducted in person by the first author. They took place at the convenience of the participants, either at their clinics or in quiet public spaces of their choice. Each interview lasted between 60 and 90 min. Interviews took place in colloquial Arabic and were recorded with the permission of the participants. They were then manually transcribed and anonymised. Coding and analysis were informed following the approach outlined by Pope and colleagues [45]: Interviews were thematically analysed through an initial line-by-line coding, followed by a focused coding to draw out themes and categories from the codes. Data collection, coding and analysis were done in an iterative manner, comparing the codes within and between interviews to make sure no more themes would arise, and identifying the core categories that structure the results section.

## Results

Health and social service providers interviewed for this study provided in-depth insight into the health and social needs of older Syrian refugees, with particular focus on non-communicable diseases and mental health problems, access to services and ageism as underlying drivers of health inequalities. They also made concrete recommendations as to how the aid response could better serve older refugees.

### Health problems experienced by older Syrian refugees

Reflecting on the health situation of their beneficiaries, Lebanese health and social workers highlighted that NCDs were the most widespread among older Syrian refugees. Leading among NCDs was diabetes, followed by blood pressure, cholesterol and heart conditions. Many older refugees also suffered from bone and joints related diseases which rendered them immobile, home bound and prone to experience isolation and loneliness. A psychologist explained, “in addition to diabetes and blood pressure, many older refugees who visit the clinic suffer from back and leg problems which confined them to home, increasing their social isolation and affecting their mental health.”

Study participants also discerned other, less prevalent, diseases among older refugees, which they believed were mainly triggered or exacerbated by refugees’ poor living conditions. For instance, pulmonary disease was connected to cold and draughty living quarters, or to the burning of tyres for heating. According to one of the nurses, “…older refugees suffer more frequently than younger refugees from pulmonary diseases in winter due to lack of heating and to overcrowding whereby viruses spread rapidly among the residents of the same tent or house.” Lice, scabies and other skin problems were also considered to be widespread among older Syrian refugees who were neglected by their families and who were unable to take care of themselves due to lack of water and sanitation. Additionally, diet and malnutrition were recognised to disproportionately affect older Syrian refugees. One GP reported,

Many of the older refugees’ physical health problems are caused by lack of hygiene, access to water, and malnutrition (…). Sometimes, 30 people live in the same tent, children and young adults go to school or to work and rarely bathe. This causes skin diseases like scabies. As for malnutrition, often a family would only have bread and tomatoes as a meal. Older adults are more sensitive to malnutrition which diminishes their immune system making them less able to fight even a simple flu or diarrhoea.

Health and social workers also emphasised mental health and psychosocial problems experienced by older Syrian refugees. It was considered that these rates were significantly higher among older refugees compared to other age groups receiving care at their facilities. Study participants estimated that more than 50% of their older Syrian refugee patients suffered from depression. Other mental health issues faced by older Syrian refugees included anxiety, post-traumatic-stress disorder and psychosis. Mental health problems were believed to be co-morbidities connected to multiple physical health problems and pains. A mental health nurse said, for instance that, “physical and mental health cannot be separated as physical illnesses negatively impact on the mental health. This is particularly true in the case of older refugees who suffer from multiple chronic diseases.”

Mental health and psychosocial problems were further linked to a sense of hopelessness. Compared to younger people, older Syrian refugees were believed to have fewer opportunities and limited resources to change their situation. A counsellor reported that her patients often said, “I want to die and get done with this life, I have no hope anymore.” A psychologist exemplified the notion of hopelessness among her patients by referring to a specific case—a woman in her sixties who experienced physical and emotional abuse from her husband. The older woman believed her situation was hopeless and she felt she was unable to leave the abusive relationship, because she could not sustain herself. The psychologist explained she could neither find a job at her age, nor was she eligible to benefit from the UN cash assistance, which tends to be given to younger refugees who have work prospects. Such feelings of hopelessness and helplessness, health practitioners reasoned, led to increased social isolation of older Syrian refugees which, in turn, was believed to negatively impact on their mental and physical wellbeing.

### Healthcare and social services

Health practitioners and social workers explained that the Lebanese government does not provide health coverage for refugees. Consequently, refugees depended on international and local NGOs for their health and wellbeing. NGOs provided services at the primary healthcare level and coverage at the secondary healthcare level. Study participants viewed the service provision and the coverage as vital, but nevertheless far from filling the gap and, thus, insufficient to serve the needs of refugees, especially the older ones.

At the primary healthcare level, healthcare expenditures of registered refugees were subsidised by UNHCR. One GP explained, “UNHCR covers 85 percent of the PHC [primary health care] consultation fee, subsidises medication for registered refugees and offers free diagnostic tests for those who are above 60. This is more than the government’s coverage for Lebanese people.”

While the UNHCR’s healthcare coverage was generally seen as generous, it was also recognised as insufficient help for all older refugees considering that the poverty was so great among them. Most interviewees stated that their NGOs were often requested to step in to cover PHC expenses, especially for unregistered, older refugees who fell outside of UNHCR’s mandate, when it became clear they were unable to afford the healthcare costs. As one of the social workers explained, “We do not turn down unregistered refugees whose healthcare is not covered by UNHCR, we take them in charge.”

Besides stepping in to provide health coverage, interviewees informed us that their NGOs provided services for registered and unregistered older refugees. Services included specialised health consultations, laboratory tests, medication, dental care, health awareness raising sessions and psychological support. Two of the NGOs included in our study were trained by an international NGO who specialised in ageing and, consequently, offered more targeted services for older refugees, such as NCD programmes as well as psychosocial support programmes. In two other NGOs, case managers said they worked specifically with older refugees as part of the UN PWSN programme (Persons with Specific Needs: mentally ill, single mothers, disabled, older adults) which offers shelter, cash assistance and psychological support. However, as one of the case managers clarified, “Very often older refugees do not qualify for these assistances for failing to meet the criteria which is often linked to work prospects that older people lack.”

However, practitioners considered health and social services insufficient for older refugees at the primary level. They explained that specialist services, such as MRIs and CT scans, were unavailable in PHCs and most medications commonly needed by older refugees suffering from various comorbidities were not covered or unavailable. Other specialised services, such as dental services, had to be paid out of pocket as they were neither covered nor subsidised by UNHCR or the NGOs. This was considered hugely problematic as dental diseases were perceived widespread among older Syrian refugees, which in turn was believed to contribute to their malnutrition. In addition, it was explained that the need of older refugees for mobile clinics, mental health and psychosocial services was much greater than that which the NGOs were offering.

Speaking of mental health and psychosocial support, one social worker reflected, “The aid response priority is for physical health, medication and tests when it comes to older adults (…) whereas recreational activities are very important because older refugees are extremely isolated which leads to mental and physical challenges.” The scarcity of such programmes meant however that older refugees were less able to build social connections, get involved in the community and engage in recreational activities as they were mainly confined at home. Such confinement, in turn, was considered to increase social isolation and loneliness which were believed to be directly linked to the development of poor mental and psychosocial health.

At the secondary healthcare level, the respondents informed us that UNHCR only covered 75% of the cost of life-threatening health conditions, and that too only for registered refugees. This puts NGO workers in a difficult position as their older clientele suffered from health problems and complications which were not always immediately life threatening, but, highly debilitating. Consequently, it was difficult to provide affordable secondary healthcare to this population. Instead, study participants explained that their NGOs relied on personal contacts in hospitals or among other NGOs whom they persuaded to either provide healthcare or financial support as an act of charity to their older refugees. One GP said, for instance, that older, unregistered refugees were especially “at the mercy of (…) NGOs who would cover the cost of their secondary care or they would borrow money from family or friends.”

Vital secondary care services, such as cancer treatment, were not covered by either UNHCR or the government for refugees. A social worker stated that this “leaves older refugees begging for private donations or seeking treatment in Syria.” So, older Syrian refugees suffering from health problems, such as cancer, were often forced to go on dangerous journeys back to their war-torn country where they could access cancer treatment for free. However, such journeys were not possible for all. One nurse reported, “Often older refugees suffer mobility problems preventing them from going through illegal and dangerous pathways. In addition, the higher rates of poverty among them compared to younger refugees prevented them from being able to afford cost of transportation.”

Such missing or insufficient services were partly attributed to a lack of funding and trained staff which were directly linked to the multi-layered, age-based discrimination that will be explored in the following section.

### Ageism as an underlying social determinant of health

All study participants considered ageism as one of the major underlying drivers for the health and social challenges experienced by older Syrian refugees. Ageism, they explained, was multi-layered, playing out at the levels of aid agencies, families and individuals.

The aid sector was perceived as not attuned to the needs of older refugees’ in that programmes focused mainly on children, women (including women empowerment) and young adults when providing social support and medical care. One nurse said, “The response prioritises vaccines, children and reproductive health. We need to start looking at older refugees as a priority (…). Funds are not focused on older adults, maybe because they are not worth the investment anymore?” One social worker noted that although there are often “campaigns to provide hearing aids and glasses, [they are mainly directed at] children and young adults so they can go to school or work.” The social worker clarified that it is mostly the donors, rather than the NGOs themselves, who drive the aid agenda, as they specify not only the kind of aid they would like an NGO to offer but also the targeted population who is supposed to benefit from the donations. As older refugees are not high on the donors’ agenda, they are not perceived to be a priority or a central part of aid provision. Consequently, vital medical and assistive aids fail to be provided.

Additionally, interviewees noted that older Syrian refugees were excluded from livelihood programmes in that they were considered to be too old to earn a living no matter their mental and physical capability. One of the social workers said, “Older refugees are excluded from vocational training programmes as well as the PCAP [Protection Cash Assistance Programme] because they cannot have a plan for their life, a plan to work and to become independent (….). Our NGO tries hard to get them at least the emergency cash assistance which is given only for one month but even this assistance is often rejected for those above 60 years old.”

Ageism at the level of aid agencies was perceived as a physical barrier to healthcare access. It was explained that most NGO clinics were not age-friendly. Only two NGOs that participated in this study had developed age-friendly centres and these were the NGOs that collaborated with the INGO specialised in ageing issues. One of the nurses explained, “We refurbished all our centres to accommodate older adults: accessible toilets, ramps, comfortable waiting areas.” According to the same nurse, her NGO is one of the very few that invested in such refurbishment, not only due to lack of funding, but also because of a lack in awareness about older people’s special needs.

On the level of the family, study participants observed ageism in various forms. They reported that family members often talked down to older people. For instance, a counsellor referred to a 74-year-old refugee who had told her, “I just want to be loved, I don’t want my family to dismiss me and laugh at me when I do something wrong.” Such dismissal was considered to be directly linked to physical neglect. A nurse said that during one of her home visits she “found the older refugee sitting alone in the corner with dry food in her plate, untaken medication, uncut nails and very dirty hair.” Similarly, another nurse highlighted,

An older refugee in her early 60s was brought to our NGO by her neighbour. She was fainting due to untreated diabetes and had lice in her hair due to lack of hygiene. Her children live in Lebanon but in another village. Her neighbour, an older person herself, was the only one who took care of her, she let her stay with her until she got better and now, she went back to Syria alone.

Study participants reported that family neglect was experienced more by older women compared to older men. Women, it was explained, experienced a double jeopardy as they suffer from disadvantages accumulated over the life-course due to gender-based discrimination which, in turn, makes them more vulnerable as older refugees. One of the mental health nurses noted,

Older women refugees are more neglected than men because Syrian men are more respected by their offspring (…). This is one of the reasons why older women refugees are more prone to mental illnesses than men (…). These illnesses become even more widespread as older women refugees get older as they become more ill, less independent and less productive.

Interviewees also thought that neglect from family members constituted one of the attitudinal access barriers to healthcare for older refugees. According to them, when medication and tests had to be paid out of pocket in total or in part, families either borrowed large sums of money or, as stated by one of the social workers, “in many cases families neglected them and prioritised children’s health and needs.” Familial neglect was explained as resulting from financial pressures as well as cultural values and stigma which prevent older women especially from going to clinics on their own and older men from seeking mental health support, even when it is highly recommended by the health and social care providers.

On the level of the individual, study participants explained that ageism not only stigmatised older refugees, but led to internalisation in that older Syrian refugees displayed self-directed, age-based discrimination. In such situations, ageism was perceived to be strongly related to status loss given that traditionally older people were valued and respected in Syrian society. Attitudes towards them changed since the war, causing related insecurities and hardship due to familial stress and the inability to make ends meet. Older refugees were seen as burden rather than contributors to the social household economy, and were consequently neglected as stated in the previous section. These views, in turn, were internalised by the older Syrian refugees who often thought of themselves as a burden on their families. One counsellor said, “One of my patients, in her late 60 s, sleeps on the kitchen floor next to the stove because her daughter and son-in-law were complaining about her snoring. She just wants to make herself invisible because she feels as a burden on them.” Similarly, a case manager explained,

A refugee in her early 60s used to work and live in her own tent. The latter got burnt down, she also had complications from diabetes, blood pressure and a rat bite [her tent had neither door nor heating]. She then had to stop working and move in with her relatives. Since then she has been severely depressed feeling she is a burden. She also has to clean their house to be able to stay with them which is impacting on her physical wellbeing.

Self-directed ageism also manifested in the perception older refugees have of their own self-worth. This was especially noteworthy amongst men who, according to the participants, perceive themselves as having higher status than women, and having the financial and decision-making power. One counsellor explained,

Older [male] refugees get to Lebanon already vulnerable because they lost their status when they retired. In addition, their spouses have no sexual interest in them anymore and baby-talk them. The final knock-out was the war and becoming a refugee which completely broke their self-image. When working with them we see how hard it is to break the ageist attitude they have about themselves.

Furthermore, study participants considered that attitudinal barriers to healthcare access were mainly rooted in self-directed, age-based discrimination. Practitioners noted that older refugees often neglected their own health out of fear of becoming a burden on their families. They reported that older refugees would not stick to the prescribed treatment so they could save or stretch medication, or they would not return to the clinics for follow-up treatment or for refill of their medication. One of the psychologists reported that many of the older refugees she sees tell her, “Forget about me, treat the young. It’s too late for me, but the young have a future ahead of them.” In the interview, the psychologist interpreted such a stance as self-directed, age-based discrimination “whereby older refugees consider themselves not worthy to receive medical treatment and social support given their age.”

### Recommendations from participants for a more effective humanitarian response for older refugees

In interviews, study participants were invited to reflect upon what kinds of change would be necessary, and feasible, to address the health and social needs of older Syrian refugees under these challenging circumstances. They highlighted four key areas including (a) the importance of prioritising older refugees in the aid response, (b) training staff, (c) adopting a holistic approach to care, and (d) adopting participatory approaches to intervention and policy developments that take into account the complex and special needs of older refugees as well as their capabilities.

#### Prioritising older refugees in the aid response

Health providers explained it would be crucial to prioritise older refugees as part of the humanitarian response. According to the respondents, the lack of prioritisation of older refugees also manifests in excluding them from data collection. One of the social workers told us, “The surveys conducted by NGOs do not include older refugees. But when I see an older refugee in a tent or a house I am surveying, I would include him/her in the survey. I don’t know if other service providers do the same.” Study participants related the marginalisation and exclusion of older refugees from the aid response and needs assessments to ageism manifesting in both humanitarian and Lebanese policy making. One nurse predicted, “Older refugees will be neglected as long as there are no effective policies on ageing in Lebanon and there is no infrastructure to support older adults in general.”

#### Training of aid workers

It was suggested that this discriminatory situation could be mitigated to some extent by training staff in more effective medical, psychological and social responses when dealing with older refugees and their particular needs. A psychologist who regularly lobbied for such training said, “I always report to UNHCR that more resources and trainings are needed for and about older refugees.” Unfortunately, she explained, her requests are largely ignored. Instead, she and her colleagues are offered alternative training in “working with people suffering from gender-based violence and other vulnerable groups but we were never trained on how to best help older adults.”

Training participants thought would be needed focused on learning how to read older refugees’ body language, detect their mental health problems and diagnose physical symptoms that might manifest differently compared to other age groups. One GP exemplified this by referring to sepsis, saying that the illness “presents without fever among older adults, who also have different malnutrition and appendicitis symptoms.” Most study participants requested holistic training to better understand the complex and intertwined physical, mental and social needs older refugees presented with. One GP explained, “If I was offered appropriate training about older refugees I would be able to look holistically at their needs spearing them many referrals to specialists.”

#### Adopting an holistic approach to care

All respondents insisted on the importance of prioritising mental health support and improving the living conditions and social connectedness of older refugees to achieve better physical health outcomes. However, it was explained that such holistic approaches to care were largely under-financed in the humanitarian aid sector. A social worker stated that resources are mainly spent on “physical health, medication and tests when it comes to older adults” while their social needs were side-lined, leading to loneliness and related mental health problems. As the provision of holistic care was neither supported by the humanitarian nor the governmental sectors, older Syrian refugees were dependent on the good will of their families. A nurse criticised this situation saying, “Older adults should not only be the responsibility of the family especially that often we witness neglect on the part of the family. The government and UN should take them in charge by providing safe and healthy shelter, covering their medical expenses and offering activities to integrate them in the society.”

#### Adopting a participatory approach to aid

Health providers emphasised the importance of adopting participatory approaches that involve older Syrian refugees as active agents in needs assessment, care and social inclusion. They explained that this would require a radical shift in current perception and practice as older refugees are generally perceived to be vulnerable individuals, fully dependent on aid and assistance and therefore unable to contribute to society in any meaningful way. This, a mental health nurse said, was a misconception and went on to elaborate, “first of all, we need to do an assessment and ask older refugees themselves about their needs so that we do not offer them inappropriate services.” A case manager went even further when saying, “We should invest in the experiences and skills of older refugees. This will be of great benefit to future generations. Sadly, the most complicated cases with the least success among the four categories of PSWN are the ones with older refugees.”

In order to reverse this situation and provide more effective care to older refugees, the case manager considered that,

Aid agencies should involve older refugees in the response as they are the ones who know what they need more than we do. They should also be encouraged to play bigger roles in their communities as they are the holders of heritage and traditions and of so many invaluable crafts and skills. This will not only benefit the community but older refugees themselves reflecting positively on their mental and physical wellbeing.

Other participants cautioned that while demands for more direct participation and contribution of older refugees to their healing was important, it could not be achieved as long as the humanitarian response refused to prioritise older refugees and failed to cater to their needs in a holistic manner.

## Discussion and concluding remarks

Ageism is considered to be a global phenomenon recognised by the World Health Organisation as “an important social determinant of health that has too long been neglected” [29]. Our research among older Syrian refugees in Lebanon has shown that ageism operates on macro-, meso-, and micro-levels simultaneously.

On the macro-level, study participants made apparent that the Lebanese government, and the many humanitarian agencies operating on the ground, have neglected the needs of older refugees. There is hardly any government health coverage available for refugees [46]. Support provided by NGOs was often difficult to access and insufficient, especially for older refugees requiring more specialised services, operations, and medication to treat ageing related comorbidities. While some older refugees were able to seek charity support from NGOs or relatives, others were forced on dangerous journeys back to Syria to access vital treatment and medication for free. Mental health and psychosocial support were also considered lacking for older refugees [5]. Moreover, study participants explained that older refugees were actively excluded from livelihood programmes, vocational training and cash assistance because their age identified them as “unproductive.”

On the meso-level, ageism was experienced in community and family contexts. Experiences of discrimination and neglect made it difficult for older people to take care of themselves and their health, build social connections, and engage in family and community life. Study participants explained that largely due to financial constraints and social tension, families neglected to take their older members to doctors’ appointments and withheld vital medication to save money. Similarly, other research highlights that communal and familial neglect of older people is a critical determinant of health that can result in worse physical health and mental health outcomes, cognitive decline, increased poverty, and social isolation [2, 15–19]. In our study, participants considered older refugee women particularly affected, suffering from more neglect than men; they described these women as vulnerable because ageism followed life-long experiences of gender-based discrimination. This finding is corroborated by Massey and colleagues who write, in their systematic review about health needs of older populations in humanitarian crises, that “older age, female gender, socio-economic deprivation and rural residency were frequently associated with adverse health outcomes” [47], p. 18].

On the micro-level, study participants described ageism as “self-directed” in that older Syrian refugees were believed to internalise the stigma and marginalisation they experienced on macro- and meso-levels. They highlighted that older refugees perceived themselves a burden to their families and wider community and expendable as they were unable to contribute to family income and livelihood. Consequently, older Syrian refugees often deprive themselves of basic resources, thereby diminishing their quality of life, health and wellbeing. Our findings are in line with research led by the WHO which indicates for different societal contexts, that “the association between ageism and health outcomes was strongest for self-directed ageism” [29], p. 48]. Similarly, a study by Bai and colleagues [48] found that, in China, older adults who feel a burden to their families were at higher risk of depression and Chang et al. highlight the correlation between ageism and depression globally, and in developing countries specifically [49].

Study participants explained that ageism experienced on macro-, meso- and micro-levels can lead to complex and intertwined physical, psychological and social needs among older Syrian refugees. WHO’s report highlights similarly that “among older people, ageism is associated with poorer physical and mental health, increased social isolation and loneliness (both of which are associated with serious health problems), greater financial insecurity and decreased quality of life and premature death” [29], p. 9]. To address these interconnected problems among older Syrian refugees, participants in our study recommended prioritising older refugees in the aid response by adopting an holistic approach to care provided by properly trained staff, and involving older refugees through participatory approaches in their own health and social care.

Similar recommendations have been put forth by Hutton [11] who states that an holistic and participatory approach to aiding older, war-affected populations would need to start by recognising the capacities and contributions of older refugees to societies, not only recognising their vulnerabilities. Specifically, he calls for the participation of older refugees in programme design and implementation, starting with needs assessments. He concludes that such a participatory approach would “enable more self-sufficiency, autonomy and independence, all of which are important for physical and psychological health” [11], p. 24]. Other research has found that while older refugees are often seen as passively benefiting from assistance, they are in fact an invaluable resource to their families and communities, playing major roles in maintaining and transmitting cultural traditions, passing on skills, and resolving social conflicts [9, 31–33]. Consequently, demands are getting louder for involving older refugees in aid planning, implementation, and intervention monitoring and assessment [8, 10–12, 20, 22, 23, 30, 50].

**Fig. 1:**
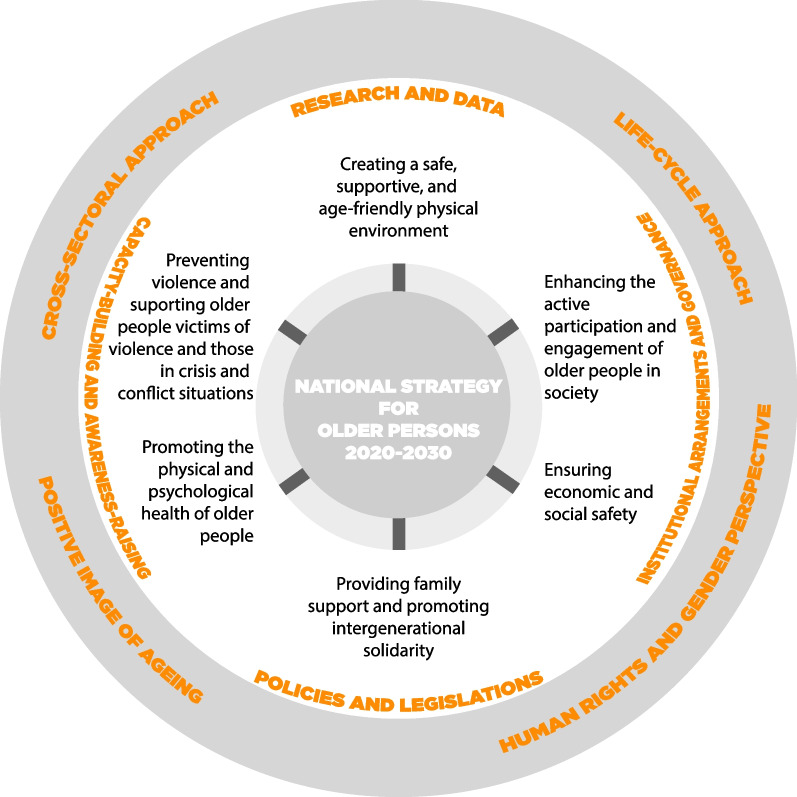
Axes and mechanisms of the National Strategy for Older Persons: Adapted from the Framework of the National Strategy for Older Persons in Lebanon 2020–2030: [51]: 22]

It is less clear how to implement a participatory approach to effectively and sustainably counter ageism and improve the quality of life and health of older refugees in different settings. Based on our findings, we believe this has to be a concerted effort, jointly driven by governmental and non-governmental decision-makers and practitioners, in order to have a deep and lasting impact. In the case of Lebanon, we suggest adapting the new “National Strategy for Older Persons in Lebanon 2020–2030” [51] to include refugees from the start. The strategy proposes a multi-sectoral plan to help empower, integrate and protect the country’s rapidly ageing population. The strategy is built on four guiding principles and several connected axes and mechanisms. The four principles are adopting a (1) human rights approach and pay particular attention to gender equality; (2) life-cycle approach which promotes safe ageing as a lifelong process; (3) cross-sectoral approach recognising that ageing is not an individual but communal responsibility that needs to be addressed and prioritised in all policies and programmes on public, private and civil society levels; and (4) positive image of ageing to change the prevailing negative stereotypes about older people. Figure 1 illustrates how the principles connect to four mechanisms of intervention along six main interrelated axes.

Considering that refugees form an integral part of Lebanon’s social fabric [40, 52, 53], we believe they should be explicitly integrated into the strategy from the start, rather than be considered a “special needs group” under the 6th axis titled “Preventing violence and supporting victims of violence and those in crisis and conflict situations” as is currently the case. This, we argue, would create the opportunity to address stigma against older refugees and positively promote their skills, provide opportunities for community participation and respect their human rights through concrete mechanisms. That is, the mechanism “Research and Data” (Fig. 1) would ensure the generation of disaggregated data to better understand the complex needs of older people, including refugees, while paying attention to their age, gender and legal status, to inform meaningful approaches to health and social support [28, 47]. Collecting such data will benefit everyone.

The mechanisms of “Institutional arrangements and governance” and “policies and legislation” could be mobilised to implement interventions on multiple levels: *at national level,* to include the protection of human rights, poverty reduction, improvement of infrastructure and age-friendly places, implementation of national policies ensuring access to life-long learning, employment, decent wages, health care with attention to age-related co-morbidities, and housing; *at service level,* to include provision of healthcare with attention to existing pluralistic medical traditions and creation of a referral system, connecting medical, psychosocial, and social services; *at community level,* to include the creation of information systems, development of participatory health and social needs assessments, and enabling community involvement; and *at household level*, to include strengthening support across extended families, attending to loss of family members, and providing a decent living wage (adapted from [54]).

To achieve this, the mechanism of “capacity-building and awareness-raising” will be required to bring the multiple societal sectors on board to make a collaborative effort to address political and social determinants affecting the health and quality of life of older refugees. It is only by promoting a positive image of ageing and raising awareness of the rights of older people, that diverse sectors such as health, urban planning, housing, education, media, and employment can come together to ensure the creation of safe, supportive and age-friendly environments; enhance participation of older people in the wider society; promote physical and mental health; and prevent violence and discrimination of older people, *no matter where they are from or what legal status they may have.*

We hypothesise that integrating older refugees into the Lebanese National Strategy for Older Persons from the start (rather than as add on) would lead to a healthier society overall. In fact, mainstreaming the refugees’ needs and rights of refugees across all six axes, with special attention to their health and social protection, would grant them their human rights, and contribute to the achievement of most of the UN Sustainable Development Goals, including poverty, hunger, health and wellbeing, education, gender equality, clean water and sanitation, work, reduced inequalities, sustainable communities, and justice. It would also allow older refugees to “contribute actively to the development of both their host society and their native countries (…), cut care costs, as well as protect the health of the resident citizens” [55].

Having said this, integrating older refugees into existing national systems and services necessitates the contribution and support of the international community. Refugees are the responsibility of all, not only the host countries, especially considering that 85% of them are in developing countries with limited infrastructures and resources [2, 56, 57]. International support and assistance is especially critical for Lebanon given the compounded crisis the country is witnessing since 2019 in the financial, economic and banking sectors [58]. Accordingly, the international community, the Lebanese government, international and local aid agencies, and academia, all have a moral responsibility to serve the needs of older refugees, combat ageism, and foster their capabilities so the older members of this displaced population do not remain invisible and at the bottom rung of the societal ladder.

This study has several limitations. Being an under-researched topic was a double edge sword: the collected data was novel and clearly filled a gap in knowledge, but the review of written sources was challenging given their paucity. In addition, statistics change frequently given the instability of the Syrian crisis and the refugee status in Lebanon [59]. Consequently, none of the interviewees had access to reliable data on older refugees that they could share with the researcher. Generalizability was another limitation. The interviews targeted a small number of NGOs, and only health and social workers. A more holistic view on the health and social challenges faced by older Syrian refugees in Lebanon would require the views of the refugees themselves, as well as local and international policy makers. In addition, the targeted NGOs provided primary healthcare only, thus access barriers to secondary and tertiary care were not duly covered. Finally, the NGOs in question work with refugees from low socio-economic status (SES) it would therefore be interesting to investigate whether or not older Syrian refugees from other SES face similar challenges.

In conclusionour findings highlight that ageism is a key determinant of health that negatively affects older Syrian refugees’ physical and mental health as well as their access to social support and health care. In order to improve health and social services for older refugees, it will be important to make them part of the solution by involving them as active agents in needs assessments, service development and delivery, programme and project evaluation. Lebanon’s “National Strategy for Older Persons” as well as programmes of international organisations should be adapted to that effect in order to address ageism directly as a key determinant of health on intersecting social, service and policy levels.

## Data Availability

Not applicable.

## Abbreviations

UNHCR: United Nations High Commissioner for Refugees
NCD: Non-communicable diseases
PHC: Primary Health Care
GP: General practitioner
SES: Socioeconomic status
MRI: Magnetic resonance imaging
CT: Computed tomography
